# Retraction: Tick-borne rickettsioses, neglected emerging diseases in rural senegal

**DOI:** 10.1371/journal.pntd.0014352

**Published:** 2026-05-26

**Authors:** The PLOS Neglected Tropical Diseases Editors

The *PLOS Neglected Tropical Diseases* Editors retract this article [1,2] due to concerns about compliance with the PLOS Human Subjects Research policy.

The Materials and Methods section of this article reports the use of blood samples collected from inhabitants of two Senegalese villages, Dielmo and Ndiop, which were taking part in a longitudinal study investigating malaria and tick-borne relapsing fever since 1990. The article states that the longitudinal prospective study that was approved by the National Ethics Committee of Senegal and the Local Ethics Committee (Marseille, France), and written individual informed consent was obtained from each participant or their legal guardians. The article also reports that a serological bank was created for this longitudinal study in 2008. Furthermore, the Materials and Methods section reports that the study reported in this article [1,2] used samples from the serological bank created for the longitudinal study, as well as samples collected from patients who came to a dispensary with a recent fever but who tested negative for malaria. Furthermore, the study used ticks collected from domestic animals in two regions in Senegal. The article does not report ethics approval reference numbers, and does not specify permits or approvals that were in place for the collection of ticks.

A representative of the Aix-Marseille Université Ethics Committee stated that their department’s investigation has not determined that the study in this article violated French regulations or international ethics rules. They confirmed that the area where the study was conducted is subject to a longitudinal study and that a dispensary was set up with the aim of studying malaria whilst providing necessary care for the local population. They also commented that the longitudinal study was authorized by the relevant ministry in Senegal, and that since the start of the longitudinal study, various research teams have intervened in the villages to carry out follow-up studies and to set up a serological bank, from which some of the samples analyzed in this article originate. Furthermore, the representative confirms that the study used arthropod vectors collected from domestic animals between November-December 2008, human samples from the serological bank collected in 2008, and samples collected during dispensary care. They also provided the documents listed below for editorial review.

- A document without a reference number issued on August 17, 1999, by the Comité d’Éthique of the Institut Pasteur de Dakar granting approval for the two studies titled:
  - *“Etude randomisée en double aveugle de la tolérabilité et de l’efficacité de l’Arstesunate plus Amodiaquine comparée à l’Amodiaquine plus placébo pour le traitement des accès de paludisme non compliqués à Plasmodium falciparum à Mlomp (Casamance), Sénégal.”*
  - *“Recherche épidémiologique pour des approches thérapeutiques nouvelles et la définition de stratégies vaccinales face au paludisme chimio-résistant.”*
- A document with reference number #00033145688929 issued on June 29, 1999, by the Comité Consultatif D’Éthique of the Faculté de Médecine et de Pharmacy of the Université Cheikh Anta Diop in Dakar, Senegal, providing approval for the collection of venous blood samples strictly limited to healthy, non-anemic individuals with good nutritional status.
- Document #969 MSPM/DS/DER issued on September 23, 2006, by the Ministère de la Santé et de la Prévention Medicale of the République de Sénégal, granting approval for a study titled “*Etude de l’histoire naturelle du paludisme: protocole d’étude mené dans les villages de Dielmo et Ndiop arrondissement de Toubacouta Région de Fatick/Senegal.*”
- Document #09–019 issued on December 9, 2009, by the Comité d’Éthique de l’Institut Fédératif de Recherche de la Faculté de Médecine de Marseille (IFR48, Marseille, France) involving a study titled “*Recherche des Rickettsioses au Sénégal*”.

PLOS reviewed the documentation provided by the institution and concluded that the documents did not fully resolve the journal’s concerns. Specifically,

- The documents issued in 1999 and 2006 appear to describe different studies than the one reported in this article [1] and they do not mention approval for the secondary analysis of these samples as part of an unrelated study. Furthermore, these documents do not mention approval for the long-term storage of these samples in a serological bank to be used in future research. Furthermore, PLOS considers it unlikely that approvals issued in 1999 would be applicable for sample collection carried out in 2008 and 2009.
- The ethics approval document N°09–019 was not issued by a local ethics body in Senegal, and was issued after the sample collection was completed. PLOS’ Human Subjects Research policy requires authors to obtain prospective ethics approval from local ethics bodies for studies involving human subjects or samples collected from human subjects. In addition, PLOS identified potential competing interests between the committee that granted the ethics approval and one or more of the article’s authors.

OM, FF, and CS did not agree with the retraction. GD, JFT, and DR either did not respond directly or could not be reached.
